# Risk factors for women’s non-utilization of decentralized primary health care facilities for postnatal care in rural western Ethiopia

**DOI:** 10.1177/2633494120928340

**Published:** 2020-06-26

**Authors:** Habtamu Tolera, Tegegne Gebre-Egziabher, Helmut Kloos

**Affiliations:** Department of Geography and Environmental Studies, Addis Ababa University, Addis Ababa, Ethiopia; Department of Geography and Environmental Studies, Wollega University, Nekemete, Ethiopia; Department of Geography and Environmental Studies, Addis Ababa University, Addis Ababa, Ethiopia; Department of Epidemiology and Biostatistics, University of California, San Francisco, San Francisco, USA

**Keywords:** decentralized health care facilities, Gida Ayana, postnatal care non-utilization, risk factor, rural western Ethiopia

## Abstract

**Objective:**

Evidence suggests postnatal care contributes to reductions in maternal mortality. In Ethiopia, the proportion of women who do not utilize postnatal care after birth is high and the frequency of postnatal checks falls short of the four visits recommended by World Health Organization. This study examined risk factors associated with non-utilization of decentralized local health facilities, namely, health posts, health centers, and a primary hospital, for postnatal care services in Gida Ayana *Woreda* in rural western Ethiopia.

**Methods:**

In this study, 454 mothers were examined for the following risk factors: *kebele* (the smallest administrative unit in Ethiopia) in which decentralized health care facilities were located, postnatal woman’s age, antenatal care service visit, experience of postnatal complications, knowledge of postnatal complications, knowledge of the recommended number of postnatal care visits, knowledge of the availability/provision of postnatal care, and health extension workers’ home visits. Bivariate and multivariable logistic regression analyses were applied to identify predictors of non-utilization of decentralized local facilities for postnatal care services.

**Results:**

Over half (55.7%) of the women did not utilize postnatal care within 42 days of delivery, and only 10.0% utilized the care considered appropriate according to World Health Organization guidelines. After adjusting for various potential confounding factors, we found the following risks to be strongly associated with non-utilization of decentralized health care facilities for postnatal care services: some outer rural administrative decentralization entities such as Angar, Lalistu, and Ejere *kebeles*; age 35 years or older (adjusted odds ratio = 3.4, 95% confidence interval: 1.4–8.3), not receiving antenatal care during this pregnancy (adjusted odds ratio = 2.0, 95% confidence interval: 1.1–3.7), no experience of any postnatal complications (adjusted odds ratio = 3.3, 95% confidence interval: 1.7–6.4), and no knowledge of at least one postnatal complication (adjusted odds ratio = 2.0, 95% confidence interval: 1.2–3.3). Risk factors highly but less strongly associated with women’s non-utilization of postnatal care services were no knowledge of the standard number of postnatal care visits recommended, no knowledge about the availability/provision of services at a local health facility, and no home visit from health extension worker by day 3 post-delivery.

**Conclusion:**

The risk factors for women’s non-utilization of decentralized health care facilities for postnatal care identified in this study need to be considered in interventions for enhancing the utilization of the service and reducing maternal and newborn deaths in rural western Ethiopia. Strengthening of postnatal care services, especially in the more remote *kebeles*, should include upgrading of the referral system and expansion of counseling of women by health extension workers.

## Background

Providing timely access to postnatal care (PNC) is one of the most effective methods of improving maternal health outcomes in less-developed countries.^1,2^ According to World Health Organization (WHO), the postnatal period begins at 1 h after the delivery of the placenta and continues until 6 weeks after delivery.^3^ PNC is important for maternal well-being; it prevents cognitive complications and illness that might result from childbirth.^4–7^ Skilled care during this period can help protect against maternal complications and deaths.^1,4,8,9^ One researcher suggested that 88–98% of all pregnancy-related deaths are preventable if postnatal women receive timely and effective PNC.^10^

Non-utilization of PNC hinders initiatives aimed at improving maternal morbidity and mortality because PNC is an essential component of those initiatives.^1,4,5,8,11,12^ A 2015 analysis of maternal mortality documented 303,000 mothers’ deaths worldwide due to pregnancy-related complications.^13^ More than two-thirds of maternal deaths occur as a result of non-utilization of PNC,^1,11^ and 62% of these deaths happen in the postnatal period.^14^ Over 50% occur on day 1.^15,16^

The maternal mortality rate (MMR) of Ethiopia is 410 per 100,000, which is higher than rates in some other low- and middle-income countries.^17,18^ It is far higher than the MMR of the United Kingdom (9 per 100,000), higher than the global average (196 per 100,000), above the Eastern sub-Saharan African average (368 per 100,000), and higher than the MMR of Kenya (338 per 100,1000).^17^ In low- and middle-income countries, the number of women obtaining PNC examinations is much lower than the number receiving antepartum and intrapartum services.^6,8,14^ This is the case in Ethiopia, where use of PNC is markedly very sluggish; even in safe motherhood programs, PNC utilization in Ethiopia is lower than use of antenatal care (ANC) and skilled health providers at birth.^11,13,19^ Only 17% of Ethiopian women receive a PNC check-up. In Oromia, the largest and most populous region and the region in which the present study was conducted, average PNC use was only 9%.^18^ This is the lowest for all regions of Ethiopia.

Research suggests the following are risk factors for women’s non-utilization of PNC: availability and accessibility of health facilities; availability of transport and rural road network; maternal literacy, age, and occupation; cultural beliefs; attitude of providers; place and mode of delivery; history of pregnancy; knowledge of pregnancy programs, complications, and PNC services; health promotion; and home visits.^5,7,20–22^ The extent to which any of these factors poses risks to women’s non-utilization of PNC services varies according to local cultural practices, geographic setting, and various barriers within specific societies.^14,23–25^

The effectiveness of the health system in Ethiopia is undermined by non-utilization of existing decentralized health care facilities (DHFs) for PNC.^26,27^ Beginning in 2002, the government demonstrated commitment to improving maternal health by offering services close to postnatal women’s homes through decentralization at *woreda* and district levels and innovative, community-based approaches.^28^ The reforms increased the number of frontline health workers, including health extension workers (HEWs); extended community-based peripheral health centers and health posts to reduce geographical barriers to reproductive health services; provided free PNC service^27^; and introduced guidelines for PNC, including adoption of the WHO recommendation that women make a minimum of four PNC visits.^29,30^ Since 2004, under these arrangements, more than 30,000 female HEWs have been trained at the national level and deployed to the frontline health posts at the *kebele* level in *woredas* all over Ethiopia, working as community mobilizers to promote maternal health and greater health in the general population.^31^ However, despite these measures, PNC utilization is still far below the standard.^3^

Recent national surveys indicate that although Ethiopia’s MMR appears to have gone down over the past decade (from 673 in 2011 to 410 in 2016), it is still high compared with some other sub-Saharan countries.^17,18^ Low PNC utilization is reflected in the high MMR, which prevented Ethiopia from achieving Millennium Development Goal 5.^19,32^ Furthermore, per the 2015 Gida Ayana *Woreda*-Based Health Sector Plan Performance Evaluation Report, Gida Ayana *Woreda* had done little to improve PNC to achieve the goal.^33^ Moreover, studies that have been conducted elsewhere in Ethiopia explicitly examined factors associated with PNC uptake in larger, urban settings of the country, not in rural areas such as Gida Ayana.^34–36^ Risk factors associated with non-utilization of DHFs for PNC service in Ethiopia have not been well addressed, and the literature says little regarding the reasons women do not seek PNC from DHFs.^3,26^

The circumstances in rural western Ethiopia and the poor documentation of risk factors in non-utilization of PNC have created a gap in the country’s ability to improve postnatal outcomes; this study was undertaken to fill this gap. The results from this study will help public health managers, practitioners, and policymakers develop interventions aimed at improving access to PNC services and thus may help reduce maternal morbidity and mortality in rural western Ethiopia.

## Methods

### Study setting and period

This study was conducted from November 2016 to January 2017 in Gida Ayana *Woreda*, rural western Ethiopia. Gida Ayana *Woreda* is located at 42 km north of Nekemte, the capital of Eastern Wollega Zone, and 440 km from Addis Ababa.^37^ The *woreda* has 28 administrative *kebeles*. The total population of the *woreda* was estimated at 140,484 in 2013; of this number, 65,556 were female population.^38^ According to the Eastern Wollega Zone Finance and Economic Development Office, 10,577 women of reproductive age (15–49 years) resided in Gida Ayana in 2015.^37^

### Study design and population

A community-based cross-sectional study design was used. The research was conducted with randomly selected mothers who gave birth in the 5 years preceding the data collection period.

### Sample and recruitment

A total sample size was determined using a single proportion formula employing a population estimate of 33.7%, 95% confidence interval (CI), a marginal error of 5%, and a design effect of 1.5.^39^ Thus, the minimum adequate sample size was determined using the statistical estimation method.^40^ As the source population was assumed to be less than 10,000, the sample size was corrected. By adding 5% for contingency, the final sample size determination was 459 women.

A two-stage sampling strategy was used to ensure representativeness of the sample. In the first stage of sampling, the four *kebeles* of Ayana, Angar, Ejere, and Lalistu were randomly selected using the lottery technique. In the second stage, households having women who had their last child during the 5 years prior to the study in the four selected *kebeles* were identified via HEWs. Then, qualified women from each *kebele* were selected based on the total number of households in each selected *kebele* (proportionate to size) using a Microsoft Office Excel–generated random number. If a household had more than one eligible woman, the mother with the most recent birth was selected. The results reported in this article are based on data from 454 women with the primary outcome of women’s non-utilization of DHFs for PNC service.

### Data collection and quality control

Data were collected from mothers at their homes through an interviewer-guided structured survey questionnaire. The questionnaire elicited data regarding socioeconomic, cultural, and demographic factors; maternal information; and women’s knowledge regarding available health facility services. For data quality control, the questionnaire was translated into the local language, Afan Oromo, and back-translated into English by blind translators to check consistency. A pilot test was conducted outside the study *woreda* (in Guto Gida) with a sample size of 10% of the study population; modifications to the questionnaire were made on the basis of the pilot test results. Data were collected by eight experienced female health professionals recruited from the study community. They were trained for 2 days on the purpose and content of the survey prior to the actual study period. Data collection was supervised on a daily basis. Every day, the completed questionnaires were cross-checked for quality and consistency. Confidentiality and privacy of every woman’s information were ensured; no identifiers of the study participants were used.

### Dependent variable

The outcome variable of this study was skilled PNC service utilization. The variable was coded y = 1 if mothers reported they did not receive PNC from skilled health personnel (midwife, nurse, medical doctor, or HEW) at the health facility or elsewhere for their recent birth; otherwise, it was coded y = 0. The non-utilization of PNC category (y = 1) was modeled.

### Independent variables

In this study, the potential determinants that pose risks for women’s non-utilization of DHFs for PNC services (see Table 1) were as follows: rural administrative *kebele* in which DHF was located; postnatal maternal age (in years); postnatal maternal marital status; postnatal woman’s literacy; postnatal woman’s occupation; average monthly household income; woman’s autonomy in making decisions about postnatal service; local community belief that postnatal visit is unnecessary; distance to postpartum service (in minutes); access to motorized transport services; availability/types of DHFs; number of children; ANC service; knowledge of complications during pregnancy, labor, and delivery; location of last childbirth; method of last child delivery; experience of postnatal complications; knowledge of at least one postnatal complication; knowledge of the recommended number of PNC visits; attending monthly women’s meetings; knowledge of availability and provision of PNC at a local facility; HEW home visit within 3 days of delivery; woman’s perception of treatment by health care providers; and infant illness.

**Table 1.** Independent risk factors.

variable	Definition of variable	Type of variable
*Kebele*	The smallest administrative decentralization entity in Ethiopia in which decentralized health systems were located. This item was categorized into four nominal variables: Ayana, Ejere, Lalistu, and Angar, the latter three being outer rural decentralization entities considered the exposure variables.	Nominal
Postnatal maternal age	A three-category variable: Less than 20 years old, 20–34 years, and 35 years or higher age groups with the latter two categories considered the exposed. It is generally recognized that older and experienced women are more likely to be non-utilizers of PNC than younger or less experienced women.	Nominal
Postnatal woman’s marital status	Maternal marital status was dichotomized into Married and then Single; Divorced or Widowed were brought together in the exposure category.	Nominal
Postnatal woman’s literacy	Literacy level was categorized as Literate (able to read and write) or Illiterate (unable to read and write); the latter was the exposure category.	Nominal
Postnatal woman’s occupation	Maternal occupation was dichotomized into Non-Housewife activities (e.g. skilled employment, small business/service, farming) brought together in the reference category and Housewife as the exposure category.	Nominal
Mean monthly income	Total household monthly income earned was made into a two-category variable: 50 $US or higher as the exposure and Less than 50 $US category.	Nominal
Woman’s autonomy of postnatal service decision	Defined as autonomy to make decisions independently and having freedom to go from home for PNC whenever she likes to. The assumption was made that women generally are looked after and follow decisions of the husband/family/elder women in their community. This item was responded to with Self or Others, which combined family members, relatives, neighbors, and traditional birth attendants, as the exposure category.	Nominal
Local community belief that PNC visit is unnecessary	This categorical variable was measured by the mother’s response to the question ‘Does the local community believe postnatal visits are unnecessary?’ Responses were dichotomized into No or Yes, with the latter as the exposure variable.	Nominal
Distance to postnatal services	Defined as walking time (in minutes) from home to the closest PNC center. This was made into a two-category variable: Less than 30 min or 30 min or higher, with the latter as the exposure category.	Nominal
Access to motorized transport services	Mothers were asked to label difficulty getting motorized transport service, including ambulance, from their home to nearest facility as Simple/Not simple, with the latter as the exposure categorical variable.	Nominal
Decentralized health care facilities visited	Type of decentralized health care facilities visited was a three-category variable: primary hospital, health center, and health post, with the latter two considered the exposure variables.	Nominal
Number of children	Defined as the number of children a woman gave birth to which is classified into three categories: fewer than 2, 2–3, and 4 children or more; the latter two were the exposure variables.	Nominal
ANC service	Assessed from the report of mothers responding that they received ANC service or did not received ANC service, with the latter as the exposure variable	Nominal
Knowledge of complications during pregnancy, labor, and delivery	Self-reported knowledge of complications during pregnancy, labor, and childbirth (e.g. bleeding, fever, prolonged labor, foul vaginal discharge, convulsion, vision problem, head ache) was dichotomized into Yes/No, with the latter as the exposure variable. Woman generally do not visit if no problems arise.	Nominal
Location of last childbirth	Place of last childbirth was dichotomized into two categories: health facility or home delivery, with latter considered the exposure variable.	Nominal
Method of last child delivery	A three-category variable: cesarean-section, instruments, and normal vaginal birth, with the latter two as the exposure variables.	Nominal
Experience of postnatal complications	Self-reported as a three-category variable: 3 or more complications, 2–3 complications, or did not experience any postnatal complications during last birth; the latter two were the exposure variable. The assumption was that women would not visit a health facility if they did not face any complications.	Nominal
Knowledge of at least one postnatal complication	Defined as knowledge of at least one complication that occurred to themselves; yes or no response, with the latter as the exposure category.	Nominal
Knowledge of the recommended number of PNC visits	Response was yes or no, with the latter as the exposure variable.	Nominal
Attending monthly mothers meeting	Response was yes or no, with the latter as the exposure variable.	Nominal
Knowledge of availability/provision of PNC	Response was yes or no, with the latter as the exposure variable.	Nominal
HEW home visit	Visit from an HEW during the first 3 days after delivery was reported as yes or no, with the latter as the exposure variable. HEWs perform home visits to postnatal women, lending support or urging them to seek postnatal services at a health facility if any problem arises.	Nominal
Woman’s perception of treatment by health care providers	Mothers reported their perception of treatment as Good, Medium, or Not Good; the latter was the exposure category.	Nominal
Severe illness of infant	Assessed as a binary variable and dichotomized into Yes/No, with the latter considered the exposure variable. It is generally recognized that women visit health centers if their infants have any severe problem.	Nominal

ANC, antenatal care; HEW, health extension worker; PNC, postnatal care.

### Statistical analysis

All the questionnaires were checked manually, coded and entered into EpiData version 3.1, and exported to SPSS Version 24.0 (SPSS; IBM Corp; USA) for analysis. The data were cleaned to check for errors and missed values and any error identified was corrected. Descriptive statistics were used to calculate the frequency distribution and proportions for categorical variables. Pearson’s correlation was used to check multicollinearity among the categorical covariates; the correlation was 0.5, which was less than 0.7; therefore, all variables were retained.^41^ The bivariate logistic regression model was applied to assess the different risk factors associated with women’s non-utilization of DHFs for PNC services. The logistic regression model for a binary outcome variable (*y* = 1 or 0) is defined as follows:

(1) ln(πi)=Pr(Yi=1)=β0+β1xi

where *x* is the single covariate of the model, *β*s are the model parameters, and *πi* is the probability of being not utilized (*y* = 1) for *i*th individual. For more than one covariate, the model is defined as follows:

(2) $$\begin{array}{l} \ln\left(\pi i\right)=Pr\left(Yi=1\right)=\\ \;\;\;\;\;\;\;\;\;\;\;\;\;\;\;\beta 0+\beta 1x1i+\cdots +\beta k\;xki \end{array}$$

where *k* is the number of covariates and the remaining terms are the same as defined above. The odds ratio can be estimated by exp(*βk*). Variables with a *p* value *<*0.3 were entered into the multivariable model.^42,43^ A *p* value *<*0.05 was considered the cutoff point for statistical significance. The degree of association between the dependent variable and the risk factors for non-utilization of decentralized primary health care facilities for PNC was assessed using crude odds ratios (CORs) and adjusted odds ratios (AORs) with 95% CI. We used before and after adjustment to ensure a reliable statistical estimate for a potential confounding variable.^44^ The Hosmer–Lemeshow test was used to compare and rule out the goodness of fit of the final models.^45,46^

#### Ethical considerations

The study was approved by the Wollega University Research Ethics Review Board (WU99529H1-3/2016), and a formal letter of permission to conduct the research was obtained from the Oromia Regional Health Bureau. All study participants were informed that they had the right to withdraw any time during the interview without giving any reason. Written consent in the form of a signature or a thumbprint was obtained from all participants or their legal guardians.

## Results

### Postnatal women’s socioeconomic, cultural, and demographic characteristics

A total of 459 mothers were contacted and 454 were included in the study, making the response rate 99.0%. Table 2 describes the background characteristics of the participants. The majority of the respondents (56.0%) were in the age range of 20–34 years; 84.0% were married. Almost half of the respondents belonged to the Oromo ethnic group and 57% were Christians. Almost half of the women were illiterate. Forty-four percent reported they were housewives or had no paid employment. Forty percent of the mothers reported monthly household income of about 1323 Ethiopian birr or less (equivalent to 49 $US or less) and 34.8% reported they were located 30 or more minutes away from the nearest local health facility. More than half of the mothers reported they considered postnatal visits unnecessary culturally, and 29.5% said their postnatal health care decisions were made by others, specifically their family members, relatives, or neighbors.

**Table 2.** Socioeconomic, cultural, economic, and demographic backgrounds of participants (*N* = 454).

Variable	Frequency (*n*)	Percent (%)
Postnatal women’s age in years		
19 or younger	127	28
20–34	255	56.2
35 or older	72	15.9
Postnatal women’s marital status		
Married	381	83.9
Single/divorced/widowed	73	16.1
Postnatal women’s ethnicity		
Oromo	222	48.9
Amhara	144	31.7
Tigre	88	19.4
Postnatal women’s religion		
Christian	260	57.3
Muslim	194	42.7
Postnatal women’s literacy		
Literate	230	50.7
Illiterate	224	49.3
Postnatal women’s occupation		
^a^Non-housewife	256	56.4
Housewife	198	43.6
Mean monthly household income		
50 $US or more	273	60.1
Less than 50 $US	181	39.9
Distance to postnatal services		
Less than 30 min	296	65.2
30 min or more	158	34.8
Access to motorized transport services		
Simple	371	81.7
Not simple	83	18.3
Postnatal women’s residence		
Urban	254	56
Rural	200	44.1
Local community believes postnatal visits are unnecessary		
No	46	54.2
Yes	208	45.8
Autonomy of postnatal service decision		
Self	320	70.5
^b^Others	134	29.5

$US: United States dollars with the exchange value of 27 Ethiopian Birr (November 2016).

a Non-housewife activities include skilled employment, small business/service, and farming.

b Others include family members, relatives, neighbors, or traditional birth attendants.

### Reproductive history and knowledge of maternal health services

The data regarding participants’ reproductive and obstetric history and knowledge about maternal health services use are given in Table 3. Almost half (47.6%) of the participants had a first-birth-order child, 65.0% received ANC service at least once from skilled health personnel during the course of their pregnancy, and 56.6% delivered at a local health facility. Fifty-three percent of the women had good knowledge of the obstetric complications associated with pregnancy, delivery, and post-delivery; 48.0% reported they did not experience any postnatal complications; 56.0% had knowledge of at least one postnatal complication; 62.0% knew the standard number of PNC visits; and 68.7% reported they never attended a monthly pregnant-women’s meeting in their *kebele.* About three-fourths of the respondents reported they knew of the availability/provision of PNC services at a local health facility. Two hundred sixty-four (58.0%) of the postnatal women reported they were not visited by HEWs during the first 3 days after delivery.

**Table 3.** Reproductive characteristics and knowledge of maternal health care services of participants (*N* = 454).

Variable	Frequency (*n*)	Percent (%)
Number of children born		
Fewer than 2	216	47.6
2–3	149	32.8
4 or more	89	19.6
ANC service		
Received	294	64.9
Not received	160	35.2
Location of last childbirth		
Health institution	257	56.6
Home	197	43.4
Knowledge of pregnancy, labor, and delivery complications		
Yes	241	53.1
No	213	46.2
Method of last child delivery		
Cesarean	39	8.6
Using instruments	61	13.4
Normal vaginal delivery	354	79.0
Experience of postnatal complications		
3 or more	101	22.3
1–2	134	29.5
No complications	219	48.2
Knowledge of at least one postnatal complication		
Yes	254	55.9
No	200	44.1
Knowledge of the recommended number of PNC visits		
Yes	280	61.7
No	174	38.3
Knowledge of the availability/provision of PNC		
Yes	335	73.8
No	119	26.2
Attended monthly women meetings		
Yes	142	31.3
No	312	68.7
HEWs home visit during the first 3 days after delivery		
Yes	190	41.9
No	264	58.2
Perception of treatment by health care providers		
Good	105	23.1
Medium	233	51.3
Not good	116	25.6
Severe infant illness during postnatal period		
Yes	246	54.2
No	208	45.8

ANC, antenatal care; HEW, health extension worker; PNC, postnatal care.

#### Service utilization by DHF type and *kebele*

The proportion of women who did not use any PNC services was 55.7%, and the proportion of those who had at least one PNC visit was 44.3%. Ninety percent of the women made fewer than four PNC visits; only 10.0% made the recommended number of four PNC visits during the postnatal period (Table 4). Among the total PNC attendants, 9.5% received postnatal services from a hospital/clinic, 55.0% from a health center, and 35.3% from a health post. Smaller proportions of women in Ejere (17%) and Angar (16%) *kebeles* utilized postnatal services at health facilities than in Ayana (37%) and Lalistu (29.5%) *kebeles*.

**Table 4.** Participants’ utilization of postnatal services by *kebele* and number of visits (*N* = 454).

Variable	Frequency (*n*)	Percent (%)
PNC use by *kebele* with a decentralized primary health care facility		
Ayana	75	37.3
Ejere	34	16.9
Angar	32	15.9
Lalistu	60	29.9
Number of visits for postnatal care		
1	119	59.2
2	38	18.9
3	24	11.9
4 or more	20	10.0
No visit	253	55.7

**Figure 1.**
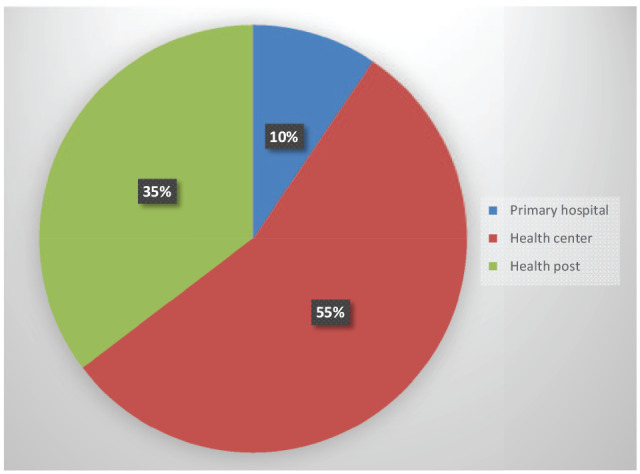
Distribution of PNC utilization by decentralized maternal health facilities visited (*n* = 201).

Among the 201 PNC attendants, 9.5% received postnatal services from a hospital/clinic, 55.0% from a health center, and 35.3% from a health post (Figure 1).

## Risk factors for women’s non-utilization of DHFs for PNC

Table 5 shows the unadjusted and AORs for the risk factors associated with non-utilization of DHFs for PNC visits. The adjusted logistic regression analysis showed that eight risk factors were significantly associated with non-utilization of DHFs for PNC services: location of DHFs in outer rural *kebeles*, older age, no ANC visit, no experience of postnatal complications, no knowledge of postnatal complications, no knowledge of availability/provision of PNC at a local facility, lack of knowledge of recommended number of PNC visits, and no home visits by HEWs to lend support to postnatal women.

**Table 5.** Risk factors associated with women’s non-utilization of DHFs for PNC services in Gida Ayana *Woreda*, rural western Ethiopia (*N* = 454).

Variable	Postnatal care service		UOR (95% CI)	AOR (95% CI)^a^
	Non-users *n* (%)	Users *n* (%)		
*Kebele* in which decentralized health care facilities were located				
Ayana	19 (20.2)	75 (79.8)	1	
Ejere	43 (55.8)	34 (44.2)	4.9 (2.5–9.8)	7.8 (3.2–18.9)**
Lalistu	91 (74.0)	32 (26.0)	11.2 (5.8–21.3)	12.0 (4.9–28.9)**
Angar	100 (62.5)	60 (37.5)	6.5 (3.6–11.9)	12.4 (5.1–30.2)**
Postnatal women’s age (in years)				
Less than 20	56 (44.1)	71 (55.9)	1	1
20–34	149 (58.4)	106 (41.6)	1.7 (1.1–2.7)*	2.1 (1.1–3.6)*
35 or higher	48 (66.7)	24 (33.3)	2.5 (1.3–4.6)*	3.4 (1.4–8.3)*
Postnatal maternal marital status				
Married	214 (56.2)	167 (43.8)	1	
Single/divorced/widowed	39 (53.4)	34 (46.6)	0.8 (0.5–1.4)	
Postnatal women’s literacy				
Literate	111 (48.3)	119 (51.7)	1	1
Illiterate	142 (63.4)	82 (36.6)	1.8 (1.2–2.7)*	1.3 (0.7–2.2)
Postnatal women’s occupation				
^b^Non-housewife	130 (50.8)	126 (49.2)	1	1
Housewife	123 (62.1)	75 (37.9)	1.5 (1.1–2.3)*	1.0 (0.6–1.7)
Mean monthly household income				
50 $US or more	141 (141)	132 (48.4)	1	1
Less than 50 $US	112 (61.9)	69 (38.1)	1.5 (1.1–2.2)*	1.4 (0.8–2.3)
Autonomy of postnatal service decision				
Self	174 (54.4)	146 (45.6)	1	1
^c^Others	79 (59.0)	55 (41.0)	1.2 (0.8–1.8)***	1.1 (0.6–1.9)
Local community’s cultural beliefs that PNC is unnecessary				
No	152 (61.8)	94 (38.2)	1	1
Yes	101 (48.6)	107 (51.4)	0.5 (0.4–0.8)	0.8 (0.4–1.3)
Distance to postnatal services				
Less than 30 min	154 (52.0)	142 (48.0)	1	1
30 or more minutes	99 (62.7)	59 (37.3)	1.5 (1.1–2.2)*	1.2 (0.6–2.2)
Access to motorized transport services				
Simple	210 (56.6)	161 (43.4)	1	
Not simple	43 (51.8)	40 (48.2)	1.2 (0.7–1.9)	
Availability/type of decentralized health care facilities visited				
Primary hospital	17 (47.2)	19 (52.8)	1	
Health center	96 (46.4)	111 (53.6)	0.5 (0.2–1.1)	
Health post	140 (66.4)	71 (33.6)	0.4 (0.2–1.0)	
Number of children woman had				
Fewer than 2	128 (59.3)	88 (40.7)	1	1
2–3	71 (47.7)	78 (52.3)	0.6 (0.4–0.9)	0.7 (0.3–1.2)
4 or more	54 (60.7)	35 (39.3)	1.0 (0.6–1.7)	0.8 (0.3–1.6)
ANC service				
Received	143 (48.6)	151 (51.4)	1	1
Not received	110 (68.8)	50 (31.2)	2.3 (1.5–3.4)**	2.0 (1.1–3.7)*
Knowledge of complications during pregnancy, labor, and delivery				
Yes	133 (55.2)	108 (44.8)	1	
No	120 (56.3)	93 (43.7)	1.0 (0.7–1.5)	
Location of last childbirth				
Health institution	134 (52.1)	123 (47.9)	1	1
Home	119 (60.4)	78 (39.6)	1.4 (0.9–2.0)***	0.5 (0.2–1.9)
Method of last child delivery				
Cesarean-section	15 (38.5)	24 (61.5)	1	1
Instruments	28 (45.9)	33 (54.1)	1.3 (0.5–3.0)	1.9 (0.6–6.2)
Normal vaginal birth	210 (59.3)	144 (40.7)	2.3 (1.1–4.6)*	2.6 (0.9–7.6)
Experience of postnatal complications				
3 or more complications	53 (52.5)	48 (47.5)	1	1
1–2 complications	50 (37.3)	84 (62.7)	0.5 (0.3–0.9)*	0.7 (0.4–1.5)
No complications	150 (68.5)	69 (31.5)	1.9 (1.2–3.1)*	3.3 (1.7–6.4)**
Knowledge of at least one postnatal complication				
Yes	121 (47.6)	133 (52.4)	1	1
No	132 (66.0)	68 (34.0)	2.1 (1.4–3.1)**	2.0 (1.2–3.3)*
Knowledge of the recommended number of PNC visits				
Yes	133 (47.5)	147 (52.5)	1	1
No	120 (69.0)	54 (31.0)	2.4 (1.6–3.6)**	2.7 (1.5–4.7)**
Attending monthly women meeting				
Yes	72 (50.7)	70 (49.3)	1	1
No	181 (58.0)	131 (42.0)	1.3 (0.9–2.0)***	0.9 (0.5–1.5)
Knowledge of the availability/provision of PNC services				
Yes	157 (46.9)	178 (53.1)	1	1
No	96 (80.7)	23 (19.3)	4.7 (2.8–7.8)**	3.2 (1.7–5.9)**
HEW home visit during the first 3 days after delivery				
Yes	82 (43.2)	108 (56.8)	1	1
No	171 (64.8)	93 (35.2)	2.4 (1.6–3.5)**	2.5 (1.5–4.2)**
Women’s perception of treatment by health care providers				
Good	65 (61.9)	40 (38.1)	1	
Medium	122 (52.4)	111 (47.6)	0.6 (0.4–1.1)	
Not good	66 (56.9)	50 (43.1)	0.8 (0.4–1.3)	
Severe illness of infant during postnatal period				
Yes	138 (56.1)	108 (43.9)	1	
No	115 (55.3)	93 (44.7)	0.9 (0.6–1.4)	

$US: United States dollars with the exchange value of 27 Ethiopian Birr (November 2016).

ANC, antenatal care; AOR, adjusted odds ratio; HEW, health extension worker; PNC, postnatal care; UOR, unadjusted odds ratio.

a Adjusted risk of the following independent variables: administrative *kebeles* in which decentralized facilities were located, postnatal woman’s age at her last birthday (years), postnatal woman’s literacy, postnatal woman’s occupation, mean monthly household income ($US), autonomy of postnatal service decision, believe postnatal visit is unnecessary, distance to postnatal service (minutes), number of children, ANC service, attending monthly mothers’ meeting, knowledge of the availability/provision of postnatal service, HEW home visit, knowledge of at least one postnatal complication, knowledge of recommended number of postnatal visits, experience of postnatal complications, location of last childbirth, method of last child delivery.

b Non-housewife activities include skilled employment, small business/service, and farming.

c Others includes family members, relatives, neighbors, or traditional birth attendants.

1 indicates the reference variable.

* *p* < 0.05; **p < 0.001; ***p ⩽ 0.3.

The odds of not utilizing PNC were significantly lower in Ayana than in the other three *kebeles* (Table 5). The postnatal women who lived in outer rural areas in which DHFs were located, specifically Angar (AOR = 12.4; 95% CI: 5.1–30.2), Lalistu (AOR = 12.0; 95% CI: 4.9–28.9), and Ejere (AOR = 7.8; 95% CI: 3.2–18.9), were more likely not to utilize PNC services compared with women in Ayana *Kebele*. Among the other socio-demographic determinant risk factors, maternal age was significantly associated with non-utilization of PNC. Women aged 20–34 years (AOR = 2.1; 95% CI: 1.1–3.6) and 35 years or older (AOR = 3.4; 95% CI: 1.4–8.3) tended not to utilize PNC visits during their postnatal course compared with younger women (age 15–19 years).

Non-utilization of PNC services was significantly higher among illiterate women (AOR = 2.0; 95% CI: 1.1–3.7). Women who did not experience any postnatal complications (AOR = 3.3, 95% CI: 1.7–6.4) were more likely to not utilize PNC services than their counterparts who had experienced at least one complication. No knowledge of postnatal complications (AOR = 2.0; 95% CI: 1.2–3.3**)**, no knowledge of the recommended number of PNC visits (AOR = 2.7; 95% CI: 1.5–4.7), no knowledge of the availability/provision of PNC services in a local facility (AOR = 3.2, 95% CI: 1.7–5.9), and no home visits by an HEW (AOR = 2.5; 95% CI: 1.5–4.2) were also significantly associated with non-utilization of PNC.

### Goodness of fit of the model

The –2 log likelihood statistic was 623.409. The statistic for the model that had only an intercept was –2LLo = 426.175. The inclusion of the parameters reduced the –2 log likelihood statistic by 218.258, which is reflected in the model chi-square for the omnibus test and the *p* value less than 0.05. Hence, an omnibus test showed the fit is adequate. This means that at least one of the predictors is significantly related to the response variable. The Nagelkerke *R*^2^ was 51.1%, indicating the explanatory variable was useful in predicting women’s non-utilization of PNC in the study area. The Hosmer–Lemeshow goodness of fit test statistic was not significant in this study, *p* = 0.806 > 0.05, suggesting that the model fits the data well. Multicollinearity in the final model was detected by examining the standard error for the coefficients. Standard errors larger than 2.0 indicate problems of multicollinearity among the independent variables.^42^ In this study, the values were less than 2.0, demonstrating the absence of multicollinearity in the developed model.

## Discussion

This study investigated women’s non-utilization of DHFs for PNC services in a rural area in western Ethiopia. In Gida Ayana *Woreda*, the main factors that pose risks for non-utilization of PNC services at the micro level were the remote rural administrative *kebeles* in which DHFs were located, older age, absence of visits to ANC service, absence of postnatal complications, lack of knowledge of postnatal complications, lack of knowledge of the standard number of PNC visits recommended, lack of knowledge of the availability and provision of PNC services at local health facilities, and failure of HEWs to make home visits within 3 days of delivery. In the study area, 55.7% of the study population received maternal health care without PNC services.

Recent studies in other countries found variations in the geographical location of the homes of mothers to be a potential and actual risk factor in non-utilization of maternal health services,^7,12^ a finding corroborated by our study. Our study also demonstrated that significant variations in the utilization of PNC services across *kebeles* in which decentralized primary health care facilities were located persisted after adjusting for covariates. Rural study populations in the outer *kebeles*, namely, Ejere, Lalistu, and Angar administrative areas, remained highly disadvantaged with higher odds of not utilizing PNC services compared with women in Ayana *Kebele*. Several studies suggested that non-utilization of PNC services, especially among rural women, may be attributed to the lack of these services or difficulty accessing them due to poor infrastructure in some rural locations.^2,7^

Another significant finding concerns the age of study participants in rural western Ethiopia. Our study revealed that non-utilization of maternal PNC services was 2.1 and 3.4 times higher among women aged 20–34 years and 35 years or older, respectively, compared with postnatal women aged 19 years or younger. Studies in rural Indonesia, rural Nigeria, and rural South Sudan reported that age affects maternal health care utilization behavior of reproductive-age women.^7,12,47^ A study conducted elsewere noted that age of postnatal mothers at childbirth significantly affected non-utilization of PNC services.^7^ Several studies in rural areas of low- and middle-income countries reported that older and experienced postnatal mothers used post-delivery services less frequently than mothers who were younger at the birth of their children.^8,12,15,25^

In rural western Ethiopia, non-utilization of PNC services was significantly associated with non-utilization of ANC clinics during the pregnancy of the mother’s last birth. Our study found the odds of non-utilization of PNC services were higher among women who had never visited health facilities for ANC services than among those who had, corroborating the findings of other studies.^4,7,16,20,34^ Similarly, recent demographic health surveys across African countries indicate that rural postnatal residents who were ANC non-users had higher odds of not receiving postnatal services.^5^

Furthermore, our study revealed that women who had experienced postnatal complications after their last birth were much more likely to visit a reproductive health facility for PNC services than those who had not encountered any complications. Several other studies reported that non-utilization of PNC services was significantly higher among mothers who did not face any postnatal complications.^7,14,48^ Studies carried out elsewhere found that absence of postnatal complications cannot protect against either actual or potential postnatal morbidity and maternal deaths.^14,20,34,48^

In rural western Ethiopia, non-utilization of PNC services was consistently higher among mothers who had no knowledge of at least one postnatal-related complication than among their counterparts who knew about post-delivery complications. Mothers who were not aware of at least one maternal complication that can occur during the postnatal period were 2.0 times less likely to use PNC services than mothers who were aware of the potential for complications. This finding is consistent with those of other studies.^2,24,47,49–51^

We also found non-utilization of PNC services higher among women who reportedly were not aware of the recommended number of PNC visits. Mothers who did not know the recommended number of PNC visits were 2.7 times less likely to utilize PNC services than mothers who knew this information. This may be explained by the role of knowledge in increasing awareness of basic health services and health risks, leading to improved health-seeking behavior. This finding is in agreement with results from various developing countries^15,16,20,51,52^ and from other studies in rural Ethiopia.^14,48,53^

The multivariable logistic regression analysis showed that lack of awareness of the availability/provision of PNC services in a local facility was a major factor in the women’s non-utilization of PNC service. Woman who had no information about their local health facilities missed the opportunity to be informed about types, benefits, and availability of PNC services. The provision of services alone, without the communication of that provision, especially among rural residents, did not improve PNC services-seeking behavior of the study participants. This result corroborates the results of several other similar studies.^4,14,48^

The absence of home visits and counseling in remote rural *kebeles* to urge mothers to obtain PNC from DHFs was another significant factor in non-utilization of these services. The odds of not receiving PNC were 2.5 times higher among women who were not visited by a HEW than among women who were visited within 3 days of delivery. This result is consistent with research in three rural districts of Indonesia that found that the lack of visits by facility providers led to rural women’s non-utilization of PNC.^25^ The odds of receiving post-delivery care in India were 1.4 times higher among women visited by skilled professionals than among mothers who were not visited^54^; low knowledge and inadequate visits or counseling sessions at home for postnatal mothers were risk factors in non-utilization of PNC services. Similar studies in rural India and Indonesia found home visits by health personnel to be essential not only for utilization of maternal health care services among rural populations,^25,54^ but also for quality PNC content.^50,53,55^

The cross-sectional design of this study measured exposure and outcome simultaneously. The determination of causal relationships between the proposed predictors and the outcomes of interest would have been strengthened with longitudinal information. Moreover, the long recall period may have introduced information bias. The CIs were, however, too wide to consider administrative decentralization entity in particular as an important predictor of non-utilization of DHFs for PNC in this case. Hence, a larger sample size may prove helpful in subsequent studies. Despite its limitations, this study sheds light on overlooked risk factors associated with non-utilization of a decentralized health care service system for PNC.

## Conclusion

This study demonstrates that the utilization of PNC in Gida Ayana *Woreda* is still low. The identified risk factors for non-utilization need to be considered by health planners and administrators in expanding the maternal health care program in Gida Ayana and in other *woredas* in western Ethiopia. Improvements must include the strengthening of PNC and ANC services, especially in outlying *kebeles*, to bolster their referral capacity and ensure HEWs provide women with adequate counseling during home visits after delivery about the need for and the availability of PNC. Further spatial and temporal studies are required to examine distance and time barriers to ANC and PNC accessibility and utilization at the household level.

## Supplemental Material

Sup_1_Questionnaire_used_for_the_study_xyz3230633628d42 – Supplemental material for Risk factors for women’s non-utilization of decentralized primary health care facilities for postnatal care in rural western Ethiopia Click here for additional data file. Supplemental material, Sup_1_Questionnaire_used_for_the_study_xyz3230633628d42 for Risk factors for women’s non-utilization of decentralized primary health care facilities for postnatal care in rural western Ethiopia by Habtamu Tolera, Tegegne Gebre-Egziabher and Helmut Kloos in Therapeutic Advances in Reproductive Health
