# Supplementary material for: Risk factors for women’s non-utilization of decentralized primary health care facilities for postnatal care in rural western Ethiopia
Source: Ther Adv Reprod Health. 2020 Jun 26;14:2633494120928340. doi: 10.1177/2633494120928340 (PMC7323273; doi:10.1177/2633494120928340)
Supplement: Sup_1_Questionnaire_used_for_the_study_xyz3230633628d42 – Supplemental material for Risk factors for women’s non-utilization of decentralized primary health care facilities for postnatal care in rural western Ethiopia [file Sup_1_Questionnaire_used_for_the_study_xyz3230633628d42.pdf]

Household Survey Questionnaire for women
Addis Ababa University
College of Social Sciences

Questionnaire on assessment of risk factors associated with non-utilization of a decentralized health facility for postnatal care service in Gida Ayana *Woreda*, western Ethiopia: a community based cross sectional study, Oromia Region, western Ethiopia.

Consent form that certify the respondent's agreement before the interview

1. Name of the *Kebele*:____
2. Questionnaire Identification Number:_________________________

Introduction

Good morning, (or Good afternoon). My name is _____________________. I am public health science professional and now I am collecting data from pregnant women of reproductive age groups (15- 49 years) for the research being conducted to identify risk factors associated with non-utilization of a decentralized health facilities for postnatal care service in Gida Ayana *Woreda*, western Ethiopia: a community based cross sectional study by Mr. Habtamu Tolera, and Professor Tegegne Gebre Egziabher from Addis Ababa University Social Sciences College and Dr. Helmut Kloos from California University, Department of Epidemiology and Biostatistics. You are selected to be one of the participants in the study by chance. The study will be conducted through interview. Your name and other personal identifiers will not be recorded on data collection format and the information that you give us will be kept confidential and will also be used for this study purpose alone. A code number will identify every participant and no names will be used. If a report of the result is published,

only summarized information of the total group will appear. The interview takes 30 minutes on average and is voluntary and you have the right to participate, or not to participate or to refuse at any time during the interview. You will not face any problem if you do not agree to the information to be asked. Your participation on this study helps to improve and identify risk factors associated with non-utilization of a decentralized health facilities for postnatal care service in Gida Ayana *Woreda*, western Ethiopia: a community based cross sectional study, western Ethiopia. If you have any questions about this study you can ask me or contact the principal investigator Mr. Habtamu Tolera

Email Address: habtol@yahoo.com.

Mobile: +251912015545

P.O.Box:1176 or 395

Are you willing to participate in the study?

1. Yes 2. No

Interviewer who certified that the informed consent has been given in written from the respondents

Name: ___________________ Signature:___________________________Date:________________

Result

1. Completed
2. Partially completed
3. Refused to complete

Checked by:

Name: ________________________________Signature:___________________Date:__________

Instruction: For the questions that have alternatives, encircle to the response of the mother. Write appropriate response (s) on the space provided for questions for which alternatives are not given.

Thank you in advance for your cooperation!

S/N	Questions	Response option	Skip
**I**	**Socio-cultural and demographic characteristics**		
401	Postnatal woman's age at last birth	Age in complete years [_____________]	
402	Postnatal woman's marital status	Single------------------------------------0<br>Married ---------------------------------1<br>Divorced--------------------------------2<br>Widowed---------------------------------3<br>Other [specify] -------------------------99	
403	Postnatal woman's ethnicity	Oromo-----------------------------------0<br>Amhara ----------------------------------1<br>Tigre-------------------------------------2<br>Other [Specify]------------------------99	
404	Postnatal woman's religion	Protestant-------------------------------0<br>Orthodox---------------------------------1<br>Muslim----------------------------------2<br>Catholic---------------------------------3<br>Other [Specify] -----------------------99	
405	Postnatal woman's literacy level	Cannot read and write -------------------0<br>Able to read and write ------------------1<br>Primary school [1-8] --------------------2<br>Secondary school [10-12] --------------3<br>College diploma and above ------------4	
406	Estimated average household monthly income	in Ethiopian Birr:[________________]	
407	What is postnatal woman's occupation during last birth?	Housewife---------------------------------0<br>Government Employed. ----------------1<br>Small business/service------------------2<br>Farmer------------------------------------3<br>If other [specify]------------------------99	
408	What is your husband's usual working status?	Farmer-------------------------------------1<br>Merchant----------------------------------2<br>public employee/private employee-----3<br>If other [specify]-------------------------99	
409	How long did it take for you to get from your home to the closest facility on foot?	In hours/minutes: [_______________]	
410	How did you judge the availability of motorized transport service during your last labor?	Simple -------------------------------------0<br>Not simple---------------------------------1	

411	Location of postnatal woman's residence?	Urban---------------------------------------0<br>Rural--1	
412	Does the local community believe that postnatal visits are unnecessary?	Yes--0<br>No---1	
**II**	**Obstetric history and knowledge level on maternal health services**		
501	Who do have the autonomy to make decision to go from home for PNC services whenever you like to?	Self---1<br>Husband------------------------------------2<br>With partner--------------------------------3<br>If other [specify]------------- ------------99	
502	Total number of children a postnatal woman gave birth to?	Number [___________________________]	
503	Do you have maternal visits to decentralized health facilities (DHFs) for ANC during your last delivery?	Yes--0<br>No---1<br>If other [specify]--------------------------99	
504	Where did you give birth to your last child?	Health institution--------------------------0<br>Home---------------------------------------1<br>If other [specify]--------------------------99	
505	Have you had a knowledge of pregnancy, labor, and birth complications during last birth?	Yes--0<br>No---1	
506	Mode of delivery during your last delivery?	Caesareans section-----------------------1<br>With support of medical instrument----2<br>Normal/ vaginal delivery-----------------3<br>If other [specify]--------------------------99	
507	Maternal exposure to postnatal complications during her last birth?	3 or more----------------------------------1<br>1-2 --2<br>No any------------------------------------3	
508	Do you have the knowledge of at least one postnatal complication after delivery to your last child?	Yes---0<br>No--1	
509	During your most recent delivery, were you informed at least about one possible postnatal complication a mother may face after childbirth?	Yes---0<br>No--1	
510	During your most recent delivery, did you know the definition and number of PNC visits recommended by WHO to a postnatal woman?	Yes--0<br>No---1	
511	Do you attend a monthly held pregnant women's meetings during your last pregnancy?	Yes--0<br>No---1	
512	During your most recent delivery, were you visited by health extension workers [at your home] during the first 3 days after delivery?	Yes--0<br>No---1	
513	During your most recent delivery, did have an aware of the availability/provision of PNC services in decentralized health facilities available at your kebeles?	Yes--0<br>No---1	

	levels?		
**III**	**PNC service use by type of local facility center**		
601	Postnatal woman's *kebele* (sub-district) or administrative decentralization entity in which the mother reside during last birth?	Name [_____________________________]	
602	Did you have postnatal visits to decentralized healthcare facilities (DHFs) in your localities during your last delivery?	Yes---------------------------------------0<br>No--1	
603	How many facility-based PNC visits did you attend from DHFs for your most recent birth?	Number [________________________]	
604	Time of check-up from a health professional after delivery and within 6 weeks postpartum	during the first 24 hours---------------1<br>on the third days------------------------2<br>on the seventh days---------------------3<br>on the fourteenth days-----------------4<br>On the forty-second days--------------5<br>No PNC visit----------------------------6<br>If other [specify]------------- ---------99	
605	The type of decentralized health facilities you visited for postnatal care during your delivery to your last child?	hospital---------------------------------1<br>Health center----------------------------2<br>Health post------------------------------3<br>Private Clinic---------------------------4<br>No PNC--------------------------------5<br>If other [specify]----------------------99	
606	Decentralized health facility available nearby your home during last birth?	Hospital --------------------------------0<br>Health center---------------------------1<br>Health post-----------------------------2<br>If other [specify]----------------------99	
607	Perception of quality of local service treatment by health care providers		
		Good------------------------------------1	
		Medium--------------------------------2	
		Not Good------------------------------3	
608	Infant illness during postnatal period		
		Yes------------------------------------0	
		No-------------------------------------1	

..................................END..

Thank you once again!
